# HIV Incidence in Rural South Africa: Comparison of Estimates from Longitudinal Surveillance and Cross-Sectional cBED Assay Testing

**DOI:** 10.1371/journal.pone.0003640

**Published:** 2008-11-04

**Authors:** Till Bärnighausen, Claudia Wallrauch, Alex Welte, Thomas A. McWalter, Nhlanhla Mbizana, Johannes Viljoen, Natalie Graham, Frank Tanser, Adrian Puren, Marie-Louise Newell

**Affiliations:** 1 Africa Centre for Health & Population Studies, University of KwaZulu-Natal, Durban, South Africa; 2 School of Computational and Applied Mathematics, University of the Witwatersrand, Johannesburg, South Africa; 3 DST/NRF Centre of Excellence in Epidemiological Modelling and Analysis (SACEMA), Stellenbosch University, Stellenbosch, South Africa; 4 National Institute of Communicable Diseases, Johannesburg, South Africa; 5 Centre for Paediatric Epidemiology and Biostatistics, Institute of Child Health, University College of London, London, United Kingdom; University of Cape Town, South Africa

## Abstract

**Background:**

The BED IgG-Capture Enzyme Immunoassay (cBED assay), a test of recent HIV infection, has been used to estimate HIV incidence in cross-sectional HIV surveys. However, there has been concern that the assay overestimates HIV incidence to an unknown extent because it falsely classifies some individuals with non-recent HIV infections as recently infected. We used data from a longitudinal HIV surveillance in rural South Africa to measure the fraction of people with non-recent HIV infection who are falsely classified as recently HIV-infected by the cBED assay (the long-term false-positive ratio (FPR)) and compared cBED assay-based HIV incidence estimates to longitudinally measured HIV incidence.

**Methodology/Principal Findings:**

We measured the long-term FPR in individuals with two positive HIV tests (in the HIV surveillance, 2003–2006) more than 306 days apart (sample size *n* = 1,065). We implemented four different formulae to calculate HIV incidence using cBED assay testing (*n* = 11,755) and obtained confidence intervals (CIs) by directly calculating the central 95^th^ percentile of incidence values. We observed 4,869 individuals over 7,685 person-years for longitudinal HIV incidence estimation. The long-term FPR was 0.0169 (95% CI 0.0100–0.0266). Using this FPR, the cross-sectional cBED-based HIV incidence estimates (per 100 people per year) varied between 3.03 (95% CI 2.44–3.63) and 3.19 (95% CI 2.57–3.82), depending on the incidence formula. Using a long-term FPR of 0.0560 based on previous studies, HIV incidence estimates varied between 0.65 (95% CI 0.00–1.32) and 0.71 (95% CI 0.00–1.43). The longitudinally measured HIV incidence was 3.09 per 100 people per year (95% CI 2.69–3.52), after adjustment to the sex-age distribution of the sample used in cBED assay-based estimation.

**Conclusions/Significance:**

In a rural community in South Africa with high HIV prevalence, the long-term FPR of the cBED assay is substantially lower than previous estimates. The cBED assay performs well in HIV incidence estimation if the locally measured long-term FPR is used, but significantly underestimates incidence when a FPR estimate based on previous studies in other settings is used.

## Introduction

To understand the dynamics of the HIV epidemic and to target and evaluate interventions to prevent HIV infection, estimates of HIV incidence at the population level are of prime importance. HIV incidence estimates can be obtained through repeated HIV testing of individuals in longitudinal surveillances. Such surveillances, however, are difficult to establish and expensive to maintain. Longitudinal data on HIV status are thus rarely available [1]. Alternatively, HIV incidence can be estimated from changes in HIV prevalence over time. The validity of these estimates, however, depends on assumptions about survival time distributions among HIV-positive and -negative individuals, which are commonly quite uncertain [2], [3]. Finally, HIV incidence can be measured in a single cross-sectional survey using laboratory tests which distinguish recent from non-recent HIV infections, reducing the need for both longitudinal and repeated cross-sectional measurement in order to estimate HIV incidence [1].

In recent years, a number of large-scale cross-sectional HIV serosurveys have been conducted. For instance, between 2001 and 2008, 20 demographic health surveys (DHS) in developing countries have included nationally representative HIV serosurveys [4]. A valid and affordable laboratory procedure to distinguish between recent and non-recent infections would allow estimation of HIV incidence in these cross-sectional surveys. One serological method to differentiate recent from non-recent HIV infections uses the BED IgG-Capture Enzyme Immunoassay (cBED assay), which measures the proportion of HIV-1-specific IgG out of total IgG. This proportion should increase with time after HIV seroconversion [5]. Seropositive individuals who test below a certain threshold of this proportion (the BED threshold) are classified as recently infected, while those testing above the BED threshold are classified as non-recently infected [5]. The time period following seroconversion after which infections are no longer considered to be recent (the so-called window period of the cBED assay) is usually estimated at approximately half a year [5], [6], [7].

The cBED assay has been used to estimate HIV incidence in many countries, including in Ethiopia [8], Rwanda [9], South Africa [10], [11], Uganda [12], Zambia [9], Zimbabwe [7], China [13], [14], and the United States [15], [16]. However, there has been concern that the cBED assay-based methods overestimate HIV incidence to an unknown extent because some non-recent infections are classified as recent [17]. In some individuals (so-called non-progressors) the proportion of HIV-1-specific IgG never rises above the recency threshold, and in other individuals (so-called regressors) who have been HIV-infected for a long time, the proportion may fall below the threshold after having previously progressed above it. Regression to levels below threshold can occur for a number of biological reasons that decrease HIV-1-specific IgG relative to total IgG, including viral suppression and immune reconstitution on antiretroviral treatment (ART), concurrent infections, and late-stage HIV disease [17]. It is in principle possible to account for non-recently HIV infected individuals who are misclassified as recently infected, but the HIV incidence estimates will depend on the estimate of a long-term false-positive ratio (FPR) [6], [7], [18]. All current methods for this correction effectively assume that by some finite time after HIV infection (the maximum BED progression time) all individuals, with the exception of non-progressors, will have progressed to the BED threshold [18]. From previous empirical observations, it is known that the maximum BED progression time is of the order of one year [6], [7]. Thus, the fraction of all people who have been HIV-infected at least as long as the maximum BED progression time who are below the BED threshold is the long-term FPR.

We use data from a large population-based longitudinal HIV surveillance to measure the long-term FPR in a rural African community with high HIV prevalence [19] and HIV incidence [20], and then compare HIV incidence estimates based on the cBED assay to estimates based on longitudinal HIV surveillance.

## Methods

### Setting

We used dried blood spot (DBS) specimens which were collected in the longitudinal population-based HIV surveillance conducted by the Africa Centre for Health and Population Studies (Africa Centre), University of KwaZulu-Natal [21]. The HIV surveillance area is located near the market town of Mtubatuba in the Umkhanyakude district of KwaZulu-Natal. The area is 438 square kilometers in size; it has a population of approximately 85,000 almost exclusively Zulu-speaking people who are members of about 11,000 households [22]. In 2004, the overall HIV prevalence among residents in the surveillance area was 27% in women (15 to 49 years of age) and 14% in men (15 to 54 years of age) [19]. The surveillance methods have been described elsewhere [20], [23]. Ethics permission for the HIV surveillance at the Africa Centre was obtained from the Research Ethics Committee at the College of Health Sciences, University of KwaZulu-Natal. All participants in the study provided written informed consent for the analysis of their samples.

### Samples

All women aged 15–49 years and all men aged 15–54 years who were resident in the surveillance area at the time of visit of an HIV surveillance fieldworker were eligible for HIV testing. Different samples were used for the different analyses conducted for this article. The samples for estimation of the long-term FPR consisted of cBED assay results for blood specimens contributed by individuals who tested HIV positive in the surveillance in the time period from June 2003 through June 2006. In order to be included in the sample, the specimens had to meet the following criteria. First, they were follow-up specimens from individuals who had previously tested HIV-positive in the surveillance. Second, the time period between the first positive HIV test and the follow-up specimen exceeded the maximum BED progression time. Third, the specimen was the earliest follow-up specimen that met the second criterion. Our count of long-term false-positive individuals included all individuals who were classified as recently HIV-infected and had been infected for longer than the maximum BED progression time, i.e. it included both non-progressors and regressors.

For the further cBED assay analyses we used a maximum BED progression time of 306 days (sample size *n* = 1,065) as baseline assumption. In order to assess the sensitivity of the long-term FPR to the assumed maximum BED progression time, we varied progression time length from 250 to 400 days in daily intervals. Table 1 shows sample size and the number of individuals who were falsely identified as recently HIV-infected for the BED progression times when the long-term FPR reaches its maximum and minimum and for all progression times in ten-day intervals from 250 to 400 days.

**Table 1:** Long-term FPR.

Maximum BED progression time	Sample size	Number of individuals with false-positive cBED assay results	Long-term FPR (ε_2_)	
(in days)	(individuals)	(individuals)	Mean	95% CI
250	1100	18	0.0164	0.0097–0.0257
260	1094	18	0.0165	0.0098–0.0259
270	1090	18	0.0165	0.0098–0.0260
280	1083	18	0.0166	0.0099–0.0261
290	1081	18	0.0167	0.0099–0.0262
300	1070	18	0.0168	0.0100–0.0265
**306**	**1065**	18	**0.0169**	**0.0100–0.0266**
310	1056	18	0.0170	0.0101–0.0268
320	1043	18	0.0173	0.0103–0.0271
330	1035	18	0.0174	0.0103–0.0273
340	1017	18	0.0177	0.0105–0.0278
350	991	17	0.0172	0.0100–0.0273
**360**	**936**	**17**	**0.0182**	**0.0106–0.0289**
370	818	14	0.0171	0.0094–0.0285
**374**	**789**	**14**	**0.0177**	**0.0097–0.0296**
380	773	14	0.0181	0.0099–0.0302
390	755	14	0.0185	0.0102–0.0309
400	737	14	0.0190	0.0104–0.0317

FPR = false-positive ratio, CI = confidence interval. Row in bold font shows FPR at twice the window period of 153, 180, and 187 days, respectively.

For the HIV incidence estimation based on longitudinal HIV status information, we included all individuals who tested at least twice for HIV in the period from June 2003 through June 2006 and whose first HIV test in this period was negative (4,869 individuals observed over 7,685 person-years). As in previous studies of HIV incidence based on data from longitudinal HIV surveillances [24], [25], [26], [27], [28], for the purpose of estimating exposure time, we used the mid-date between the last available negative HIV test and the first available positive HIV test as an estimate of the date of seroconversion. In addition, in order to test the robustness of the longitudinally measured HIV incidence estimates to changes in the assumption about seroconversion dates, we re-estimated HIV incidence using the most extreme assumptions about the seroconversion date that are possible given the interval-censored information on seroconversion dates. At the one extreme, we assumed that all individuals in the longitudinal sample who seroconverted did so on the day immediately after the day of their last HIV-negative test. At the other extreme, we assumed that all individuals who seroconverted did so on the day of their first HIV-positive test. Under changes in the assumption of date of seroconversion, these two extremes yield maximum and minimum estimates of longitudinally measured incidence.

For the cross-sectional cBED-based HIV incidence estimation, we used the first available HIV test for all individuals tested in the time period January 2005 through June 2006 (*n* = 11,755), i.e. the period in which all second HIV tests of the people included in the longitudinal HIV incidence analysis took place. Thus, all 4,869 individuals in the longitudinal sample are also included in the sample for the cBED assay-based analysis.

### Laboratory procedures

HIV status was determined by antibody testing with a broad-based HIV-1/HIV-2 enzyme-linked immunosorbent assay (ELISA; Vironostika, Organon Teknika, Boxtel, the Netherlands) followed by a confirmatory ELISA (GAC-ELISA; Abbott, Abbott Park, Illinois, USA) [23]. If HIV-positive status was confirmed, we used another spot from the same filter paper as used for the initial test in order to conduct the cBED assay (cEIA; Calypte® HIV-1 BED Incidence EIA, Calypte Biomedical Corporation, Maryland, USA). HIV-specific IgG were detected by the BED-biotin peptide, followed by a colour reaction with streptavidin-peroxidase. The optical density values were normalized in every run using a calibrator (normalized OD (ODn) = mean specimen OD/mean calibrator OD). Specimens with ODn less than or equal to 1.2 during an initial cBED screening test were confirmed by further cBED testing of the sample in triplicate. We took the median value of the three confirmatory test results as the final ODn value. As specified by the manufacturer, an HIV-1-positive specimen for which the cBED assay gave a final ODn of less than or equal to 0.8 was considered to be a specimen of recent HIV-1 infection. Otherwise, the specimen was classified as a non-recent infection [5].

### Statistical analysis

Different formulae that use information obtained from the cBED assay have been proposed to estimate HIV incidence from cross-sectional surveys. These formulae provide incidence estimates expressed either as a rate, *Î* *_r_*, (expressed, for instance, in number of new HIV infections per 100 person-years) [18] or as the probability that in a given year a person will acquire HIV, i.e. an incidence proportion, *Î* *_p_*, (expressed, for instance, in number of new HIV infections per 100 people per year) [6], [7]. Some of us have previously derived a formula from first principles to estimate HIV incidence based on the cBED assay [18], and have commented on the assumptions made in different formulae [29], [30]. Here, we implemented four different formulae found in the literature. The formula for HIV incidence derived by McDougal and colleagues (McDougal formula) [6] is
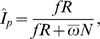
where *R* is the number of people who were classified as recently HIV-infected by the cBED assay and *N* is the number of individuals who tested HIV-negative. The mean window period of the cBED assay, 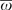, is “the mean period of time from initial seroconversion to reaching an ODn of 0.800” expressed in years in people who progress above the BED threshold [6]. The “adjustment factor”
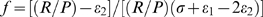
takes into account that the cBED assay does not have perfect specificity or sensitivity, *P* is the total number of people who tested HIV-positive, *σ* is the sensitivity of the cBED assay, *ε*_1_ is the short-term FPR (i.e. over the period [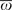, 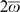]), and *ε*_2_ is the long-term FPR (i.e. over all times 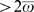). Note that the short- and long-term specificities, *ρ*_1_ and *ρ*_2_, are related to the FPRs by *ρ*_1_ = 1−*ε*_1_ and *ρ*_2_ = 1−*ε*_2_, respectively. The formula of Hargrove and colleagues (Hargrove formula) [7] is
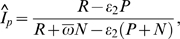
while the formula derived by McWalter and Welte (McWalter/Welte formula) [18] is
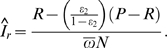



In addition, we implemented a simplified version of the McDougal formula. The adjustment factor used in the formula can be simplified to
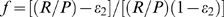
using the identity
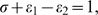
which requires no more assumptions than are used by McDougal and colleagues [29], [30].

Note that in order to implement any of the above four formulae, estimates of the long-term FPR *ε*_2_ and the window period 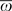 are required. For our baseline estimation, we use an 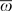 of 153 days, i.e. the window period that is recommended by the manufacturer of the commercially available cBED assay. Most previous studies reporting HIV incidence based on the cBED assay have used window periods between 150 and 160 days [6], [9], [12], [13], [14], [15], [16], [31], [32], [33], [34], [35]. A few studies have used a window period of 180 days [8], [10], [11], and a recent study from Zimbabwe calibrated a window period of 187 days in postpartum mothers enrolled in a Vitamin-A intervention trial [7]. In order to test whether our results are robust to changes in the window period estimate, we repeated our analyses with window periods of 180 and 187 days. The Hargrove and McDougal formulae require that the maximum BED progression time is twice the window period. The estimate of the long-term FPR thus depends on the choice of the window period (see Table 1).

Note also that the Hargrove, McWalter/Welte and simplified McDougal formulae do not require estimates of *σ* and *ε*_1_, which – unlike *ε*_2_ – cannot be calibrated from longitudinal data if the intervals between the last negative and the first positive HIV test in seroconverters are of the order of one year [30]. The mean period of follow-up among seroconverters in our study was 1.4 years; we thus used estimates of *σ* (0.7680) and *ε*_1_ (0.2770) from another study in order to implement the McDougal formula [6] (compare also [10]).

The McWalter/Welte formula expresses HIV incidence as a rate, i.e. as the number of HIV seroconversions per person-time at risk, while all other formulae express HIV incidence as an incidence proportion, i.e. the number of HIV seroconversions within a specified time period divided by the size of the population initially at risk. In order to directly compare all HIV incidence estimates in our study, we expressed the estimates based on the McWalter/Welte formula and the longitudinally measured HIV incidence both as rates (per 100 person-years) and as incidence proportions (per 100 people per year). We translated the rate estimates into proportions, assuming that the incidence rate, *Î* *_r_*, is constant over time *T*, by using the relationship
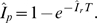



The authors of the four different formulae do not use equivalent methods for the calculation of confidence intervals (CIs). Thus, uncertainty analysis on the incidence estimates was performed as follows. Any observed proportion of HIV-negative, cBED-recent and cBED-non-recent individuals is an unbiased estimate of the underlying population proportions. Given an observed occurrence of the population proportions and the sample size, all attainable draws of the three counts can be enumerated and assigned their respective trinomial probability. Hence an exact cumulative probability distribution of attainable values of the incidence estimator can be computed. For each incidence estimate, we quote the estimator evaluated at the observed counts (the maximum likelihood estimate) and a confidence interval expressed as the central 95^th^ percentile.

To control for differences in the sex-age composition between the sample used in the longitudinal HIV incidence estimation and the sample used in the cBED assay-based estimation, we weighted the sex- and five-year age group-specific longitudinal mean incidence rates by the proportions of individuals in each of the sex-age groups in the sample used for the cBED assay-based estimation
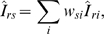
where *Î* *_rs_* is the sex-age adjusted mean incidence rate, *w_si_* are the proportions of individuals in each sex-age group in the cBED assay sample, and *Î* *_ri_* are the sex-age specific mean incidence rates. We estimated the variance of *Î* *_rs_*, var(*Î* *_rs_*), as
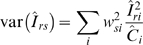
assuming that the number of HIV incident cases, *Ĉ* *_i_*, is Poisson distributed [36]. We calculated the 95% confidence limits for *Î* *_rs_* using the method based on gamma distributions described in Anderson and Rosenberg [37].

## Results

### Long-term FPR

Counting the number of DBS specimens classified as recently HIV-infected by the cBED assay in the sample of all individuals who had a previous positive HIV test more than 306 days before the date of the cBED assay-tested specimen, we obtained a long-term FPR of 0.0169 (95% CI 0.0100–0.0266). When we varied the length of the maximum BED progression time from 250 to 400 days (in daily intervals), we found that the estimate of the long-term FPR did not change significantly over the time interval, with minimum and maximum long-term FPRs of 0.0164 (95% CI 0.0097–0.0257) and 0.0190 (95% CI 0.0104–0.0317), respectively (Table 1).

### Incidence comparison

Of the 4,869 individuals included in the sample for longitudinal HIV incidence measurement, 224 people seroconverted in 7,685 person-years. Assuming that seroconversion occurred at the mid-date between the last available negative HIV test and the first available positive HIV test, longitudinally measured crude HIV incidence was 2.87 per 100 people per year (95% CI 2.53–3.27) (Table 2). Longitudinally measured HIV incidence increased to 3.09 per 100 people per year (95% CI 2.69–3.52), when we adjusted it to the age-sex distribution of the sample for the cBED assay-based incidence estimate.

**Table 2:** HIV incidence estimates.

Estimation type	Unit	HIV incidence	
		Mean	95% CI
**Longitudinal measurement**			
(7,685 person-years, 224 seroconversions)			
Crude	(per 100 person-years)	2.91	2.56–3.32
Sex-age adjusted	(per 100 person-years)	3.14	2.73–3.58
Crude	(per 100 people per year)	2.87	2.53–3.27
Sex-age adjusted	(per 100 people per year)	3.09	2.69–3.52
**cBED assay measurement**			
	(*n* = 11,755)		
*Mean of locally measured long-term FPR (ε_2_ = 0.0169)*			
McWalter/Welte	(per 100 person-years)	3.22	2.57–3.87
McWalter/Welte	(per 100 people per year)	3.17	2.54–3.80
McDougal	(per 100 people per year)	3.03	2.44–3.63
Hargrove	(per 100 people per year)	3.19	2.57–3.82
McDougal, simplified	(per 100 people per year)	3.12	2.51–3.73
*Lower bound of 95% CI of locally measured long-term FPR (ε_2_ = 0.0100)*			
McWalter/Welte	(100 person-years)	3.65	3.00–4.32
McWalter/Welte	(per 100 people per year)	3.58	2.95–4.22
McDougal	(per 100 people per year)	3.40	2.82–4.00
Hargrove	(per 100 people per year)	3.57	2.95–4.19
McDougal, simplified	(per 100 people per year)	3.52	2.91–4.14
*Upper bound of 95% CI of locally measured long-term FPR (ε_2_ = 0.0266)*			
McWalter/Welte	(100 person-years)	2.60	1.96–3.27
McWalter/Welte	(per 100 people per year)	2.57	1.94–3.22
McDougal	(per 100 people per year)	2.49	1.89–3.11
Hargrove	(per 100 people per year)	2.63	1.99–3.29
McDougal, simplified	(per 100 people per year)	2.53	1.92–3.17
*Externally measured long-term FPR (ε_2_ = 0.0560)*			
McWalter/Welte	(100 person-years)	0.65	0.00–1.33
McWalter/Welte	(per 100 people per year)	0.65	0.00–1.32
McDougal	(per 100 people per year)	0.66	0.00–1.33
Hargrove	(per 100 people per year)	0.71	0.00–1.43
McDougal, simplified	(per 100 people per year)	0.65	0.00–1.32

CI = confidence interval, FPR = false-positive ratio.

Of the 11,755 individuals included in the sample for the cBED assay-based HIV incidence measurement, 9,236 tested HIV- negative and 2,519 HIV-positive. Of the individuals who tested HIV-positive, 165 were classified in cBED assay testing as recently HIV-infected and the remainder as non-recently infected. For given *ε*_2_ and 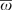, the four different formulae to calculate HIV incidence from cBED assay measurement produced very similar results. Using the baseline estimate for 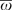 of 153 days and the locally measured *ε*_2_ of 0.0169, HIV incidence point estimates (per 100 people per year) varied between 3.03 (95% CI 2.44–3.63; McDougal formula) and 3.19 (95% CI 2.57–3.82; Hargrove formula) (Table 2). The cBED assay-based HIV incidence estimates were thus very similar in magnitude and did not differ significantly from the estimates based on longitudinal measurement (crude and sex-age adjusted) (Table 2). Furthermore, when we implemented the cBED assay formulae using the lower bound or upper bound of the 95% CI of the locally measured long-term FPR (0.0100–0.0266), the cBED assay-based HIV incidence estimates did not differ significantly from the estimates based on longitudinal measurement. By contrast, when we implemented the cBED assay formulae using the externally measured long-term FPR of 0.0560 [6], all four cBED assay-based HIV incidence estimates were significantly lower than the longitudinal estimates (Table 2).

Our finding that the cBED assay-based HIV incidence estimate was not significantly different from the longitudinal HIV incidence estimate did not change when we applied the window periods of 180 and 187 days (and their corresponding long-term FPRs of 0.0182 and 0.0177 (see Table 1)). Using the McWalter/Welte formula, the cBED assay-based HIV incidence was estimated at 2.63 per 100 people per year (95% CI 2.10–3.18) with a 180-day window period and at 2.56 per 100 people per year (95% CI 2.04–3.08) with a 187-day window period. Neither of these estimates was significantly different from the longitudinally measured HIV incidence estimates or from the cBED assay-based incidence estimates based on a 153-day window period (see Table 2).

As described above, we conducted sensitivity analysis of the longitudinally measured HIV incidence estimate by changing the assumption about seroconversion dates. Assuming that all seroconverters became HIV-seropositive on the day following the last negative HIV test, crude HIV incidence was estimated at 2.97 per 100 person-years (95% CI 2.61–3.39). Assuming, on the other hand, that all seroconverters became HIV-seropositive on the day of their first positive HIV test, crude HIV incidence was estimated at 2.85 per 100 person-years (95% CI 2.51–3.25). The longitudinal HIV incidence estimates were thus highly robust to changes in the approach to computing the seroconversion date. Even under the most extreme possible assumptions, the mean HIV incidence changed by only 2% of the estimate based on the mid-date assumption, as reported in Table 2.

When we stratified HIV incidence by sex and five-year age group (starting at 15 years of age), we found that none of the cBED assay-based sex and age-specific estimates differed significantly from the corresponding longitudinally measured sex and age-specific estimates. However, our samples in each of the sex-age groups were too small to detect significant differences with reasonable confidence. The coefficients of variation (CVs) of the sex-age specific cBED assay-based HIV incidence estimates ranged from 18% to 203%; in 13 of the 15 sex-age groups the CVs were larger than 25%; in 10 sex-age groups the CVs were larger than 50%; and in 4 sex-age groups they were larger than 100%.

## Discussion

In a rural community in South Africa, we found a long-term FPR of the cBED assay of 0.0169. This value is substantially lower than the two previous estimates of the ratio. The first estimate (0.0560) “was based on analysis of specimens from longer-term-infected individuals not known to have clinical AIDS, opportunistic infections, or to be on treatment” in the USA [6]. The article, in which this value was published, provides neither the sample size for the measurement nor the confidence limits around the estimate [6]. Thus we cannot test whether the estimate is significantly different from the value that we measure in rural South Africa. The second estimate (0.0520) was based on specimens from 2,749 postpartum mothers enrolled in a Vitamin-A intervention trial in Zimbabwe [7]. This second estimate was significantly higher than the value measured in our study (p<0.0001).

Many previous studies have used the first estimate of the long-term FPR in their estimations of HIV incidence based on cross-sectional cBED assay surveys (e.g. [9], [10], [12], [14]). In comparing cBED-based HIV incidence estimates to HIV incidence measured longitudinally in the same population, we have demonstrated that, had we used the long-term FPR of 0.0560, we would have significantly underestimated HIV incidence in this community. By contrast, using the locally measured ratio of 0.0169, we estimated an HIV incidence that does not differ significantly from the longitudinally measured incidence.

Our findings thus confirm the previous results by McDougal et al. [6] and Hargrove et al. [7] that cBED assay-based HIV incidence estimates are not significantly different from longitudinally measured HIV incidence, when a locally calibrated long-term FPR ratio is used to adjust for the imperfect long-term specificity of the cBED assay. At the same time, we have shown for the first time that the long-term FPR differs significantly across settings. Hence, results from studies that use a long-term FPR measured in another setting should be viewed with skepticism.

We further found that the different formulae to estimate HIV incidence based on the cBED assay results, did not produce significantly different values even though they differ in their underlying assumptions, suggesting that the choice of formula may not be very important for most practical purposes. Finally, we showed that the estimates of the long-term FPR based on data from a longitudinal HIV surveillance are very robust to changes in the definition of “long-term” (i.e. the choice of the maximum BED progression time).

Our longitudinal HIV incidence estimates in this article are slightly lower than previously published estimates from the same community [20], because the current study uses a sample that is different from the one used previously. In particular, unlike in the previous study, we excluded from the sample people who were identified as members of a household in the study area, but who did not themselves live in the area. We excluded this population group (which faces a significantly higher risk of HIV acquisition than household members who live in the study area [23]), because cross-sectional cBED assay surveys usually do not trace such non-resident household members.

HIV incidence estimates by sex and age group are important for validating the cBED assay method as an approach to measure HIV incidence [7], and are an important disaggregation for health policy and planning, e.g. in order to inform the targeting of HIV prevention interventions. Our current sample lacked the statistical power to meaningfully stratify the HIV incidence estimates. As more data becomes available from our site, we will in the future analyze HIV incidence across population subgroups.

The promise of the cBED assay for HIV surveillance, program evaluation and policy making, lies in the fact that it allows HIV incidence estimation from cross-sectional samples. Cross-sectional HIV status information, however, does not permit estimation of the long-term FPR, requiring researchers to obtain this parameter independently. It is thus important that the parameters necessary for HIV incidence estimation are calibrated using data from those settings where longitudinal follow-up is available. A meta-analysis of the long-term FPR of the cBED assay may help explain why the parameter estimates differ and allow the determination of valid regional parameter estimates.

It may further be necessary to measure the long-term FPR repeatedly over time. For instance, one of the reasons why people with non-recent HIV infections are falsely classified as recently infected by the cBED assay is viral suppression due to ART [38]. In October 2004, ART started to become available through the public health services in the community in which this study took place. However, only a very small number of patients received ART during the study period. By the end of December 2005, i.e. half a year before the end of the study period, approximately 500 patients received ART through the public ART programme in the district in which this study took place. Because the HIV surveillance covers less than half of the district population, we estimate that in December 2005 less than 250 people in the surveillance area were receiving ART out of a total resident population of approximately 65,000 [39]. Future studies will need to investigate whether our locally estimated cBED long-term FPR changes with increasing ART coverage.

An alternative to using the long-term FPR in order to adjust cBED assay-based HIV incidence estimates for the presence of people who are falsely classified as recently HIV-infected is to use additional information on time since seroconversion to identify these individuals and correct the misclassification. Information on time since seroconversion, which can be obtained in cross-sectional surveys, could be based on biological parameters that change with time since infection (such as CD4 count, total lymphocyte count, or viral load), clinical assessment (such as screening for HIV-related diseases that indicate late-stage HIV disease [40]), and screening for ART (through a question or laboratory test).

In conclusion, our study demonstrates that without a locally measured long-term FPR HIV incidence estimates based on the cBED assay may be severely biased, but that the cBED assay performs well in HIV incidence estimation, if a locally appropriate long-term FPR is used.
